# Exploring the association between canine perineal hernia and neurological, orthopedic, and gastrointestinal diseases

**DOI:** 10.1186/s13028-022-00655-w

**Published:** 2022-12-17

**Authors:** Tuuli Maria Åhlberg, Tarja Susanna Jokinen, Hanna Maija Salonen, Outi Maria Laitinen-Vapaavuori, Sari Helena Mölsä

**Affiliations:** Department of Equine and Small Animal Medicine, Faculty of Veterinary Medicine, University of Helsinki, Helsinki, Finland

**Keywords:** Computed tomography, Dog, Electromyography, Lumbosacral stenosis, Motor nerve conduction velocity

## Abstract

**Background:**

Perineal hernia (PH) is a relatively common condition in intact male dogs, but the etiology remains unclear. The objective of this study was to assess the contribution of gastrointestinal (GI), neurological, and orthopedic conditions to the development of PH in male dogs. Patient history with a focus on chronic GI disease was assessed using an owner questionnaire. Neurological conditions were explored, applying neurological, electromyographic (EMG), and motor nerve conduction velocity (MNCV) examinations and combining these with computed tomography (CT) imaging. To exclude possible orthopedic diseases, an orthopedic examination was conducted together with CT analysis. The chi-squared test was used to assess the associations between categorical variables.

**Results:**

Altogether, 66 male dogs with diagnosed PH were recruited for this study. The frequency of neurological, orthopedic, and GI diseases was low in dogs with PH. No signs of generalized neuro- or myopathies were detected. Still, perineal and bulbourethral reflexes were decreased or missing in 44.6% (29/65) and 40.0% (26/65) of dogs, respectively. Mild or moderate occlusion of the intervertebral foramen at the lumbosacral (LS) junction occurred in 18.5% (12/65) of dogs and was caused by spondylosis deformans in 83.3% (10/12). Moderate disc protrusion was evident in 9.2% (6/65) of dogs.

**Conclusion:**

No evidence was found that PH is caused by gastrointestinal, orthopedic, or neurological conditions. Abnormalities in perineal and bulbourethral reflexes are most likely secondary to PH.

## Background

Perineal hernia (PH) is a condition mainly affecting intact male dogs over four years of age. During the development of PH, muscles of the perineal region atrophy and separate, depleting the rectum of support and allowing pelvic organs to herniate into the perineal area [1–3]. Clinical signs include tenesmus, constipation, and pain while defecating [4]. Urinary signs are associated with bladder retroflexion [5, 6].

In previous literature, no single factor alone causing PH in dogs has been identified, implying that PH etiology might be multifactorial [2, 3, 6–9]. As it mainly affects male dogs, a hormonal component is likely, but no direct association between PH and sex hormones such as testosterone or estradiol has been identified. The role of the prostate has also been a matter of some debate [4, 7, 8, 10]. The relatively weaker pelvic structures in males compared to females may be another factor that predisposes to PH [3]. Congenital weakness of the perineal muscles and breed predisposition have also been suspected, but have not been proven [1, 4, 10, 11].

Chronic constipation and straining to defecate are thought to contribute to PH formation, especially when occurring concurrently with structural weakness of the perineal muscles [4, 12, 13]. GI diseases, such as colitis and proctitis, can cause straining, along with diarrhea, tenesmus, hematochezia, and flatulence. As these clinical signs often coincide with those of PH, an association with GI diseases has been suggested [4, 14–16]. Other diseases known to cause constipation include prostatic hypertrophy and other rarer intrapelvic masses such as tumors and paraprostatic cysts [1, 3, 17]. In addition, constipation and/or straining may be caused by anal gland impaction or sacculitis and chronic urinary tract conditions, as well as painful orthopedic and neuromuscular disorders such as diseases of the lumbosacral (LS) spine, hips, and pelvic limbs [4, 13, 18]. Studies assessing GI diseases that might contribute to PH are lacking.

Some researchers have suggested that neurological diseases may play a role in the etiology of PH in dogs, although the extent of the neurological damage, as well as its cause, is yet to be determined [2, 10]. In one study, an intraoperative electromyography (EMG) assessment of the perineal muscles of dogs with PH was conducted, revealing spontaneous activity in 35/40 dogs, indicative of denervation or primary muscle disease. The sites of possible nerve damage were localized to the pudendal nerve or the ventral rami of the sacral nerves (S1–S3), and this damage was hypothesized to be a consequence of pressure applied to nerves in the pelvic canal by a mass such as an enlarged prostate [2]. Another possible condition capable of causing nerve damage in this area is degenerative LS stenosis. This is a syndrome associated with degeneration of the structures of the LS junction, which can be associated with narrowing of the vertebral canal, intervertebral foramina, or both, and can thus potentially lead to compression of the cauda equina [19].

The objective of this prospective study was to explore possible etiological mechanisms in the development of PH in male dogs, such as neurological, orthopedic, and GI diseases. This is the most thorough study to date to prospectively assess preoperative data of dogs with PH and to apply methods not previously used to investigate PH etiology. Methods include orthopedic and neurological examinations, EMG, determination of motor nerve conduction velocity (MNCV), and computed tomography (CT), as well as an owner questionnaire. Our hypothesis was that neurological, orthopedic, and GI conditions do not contribute to the formation of PH in these dogs.

## Methods

### Inclusion criteria and ethical considerations

This prospective descriptive study included dogs with naturally occurring PH referred to the Veterinary Teaching Hospital of the University of Helsinki (VTHUH) from March 2017 to December 2020. All PH cases were confirmed at the VTHUH based on rectal palpation before being included in the study. This paper is part of a larger research project assessing the etiology and treatment of canine perineal hernia. The population of dogs in the present study also includes a cohort of male dogs from a previously published study [20]. We excluded dogs that had severe renal, hepatic, or cardiac disease or had an American Society of Anesthesiologists physical status rating of 4 or 5. Dogs that had previously undergone surgical treatment for PH were excluded.

Ethical permission for this research was provided by the Finnish national Project Authorization Board: ESAVI/4467/04.10.07/2017. Owners gave written consent for their dogs to take part in the study and had the possibility to withdraw at any time.

### Questionnaire

Upon entering the study, owners were required to complete a questionnaire, which included five sections: signalment, current GI signs, current urinary signs, history of GI signs and diseases, and history of non-GI diseases. The castration status (intact, castrated, and received hormonal treatment) and age of possible castration was also noted. The type of diet fed (commercial, home-cooked, raw, commercial and home-cooked, commercial and raw, other) before PH related clinical signs and the time since the latest deworming (months) were recorded.

Current GI signs included questions on inappetence, vomiting, diarrhea, tenesmus, defecating small quantities at a time, problems evacuating the bowel, staying in the defecating position for longer than usual, constipation, thickened stool, mucus in the stool, and blood in the stool. In addition, fecal incontinence, rectal prolapse, and swelling or bulging in the perineal area were recorded (yes/no/not sure). The duration of current GI signs associated with PH was evaluated (< 2 weeks, 2–4 weeks, > 4 weeks but < 3months, 3–6 months, > 6 months, not sure). The current urinary signs noted were pollakiuria, stranguria, dysuria, hematuria, and urinary incontinence (yes/no/not sure).

To assess the history of chronic GI signs and diseases, owners were asked to evaluate clinical signs occurring before the GI signs associated with PH that lasted for more than 3 weeks. The clinical signs asked about were vomiting, diarrhea, flatulence, blood or mucus in the feces, and constipation. In addition, owners were asked about any previous diseases diagnosed by a veterinarian such as GI diseases (colitis, proctitis, inflammatory bowel disease, food sensitivity, tylosin-responsive diarrhea), orthopedic diseases (hip joint laxity or dysplasia, pelvic or spinal fractures, degenerative changes in the lumbar vertebra or the LS junction), neurological conditions (e.g., intervertebral disc disease or a generalized neurological condition), anal sac inflammation or impaction, as well as tumors around the anus. Previously diagnosed gastrointestinal, orthopedic, and neurological diseases were categorized as “yes”, “no”, or “not sure”.

### Evaluation of dogs

The dogs underwent a physical examination and a bloodwork assessment, including a complete blood count and serum biochemistry to evaluate the eligibility for the study. These were performed by a PhD researcher in clinical veterinary medicine with a degree in veterinary medicine (TÅ). The final inclusion of dogs in the study was based on the inclusion criteria and decided by an ECVS-certified veterinary surgeon (SM), who also conducted the orthopedic examinations. The neurological examination and the EMG and MNCV studies were conducted by an ECVN-certified veterinary neurologist (TSJ). The dogs were anesthetized and EMG, MNCV, and CT studies were conducted, after which they underwent PH surgery, including prescrotal castration if intact. CT images were analyzed by a veterinarian experienced in diagnostic imaging (HS).

### Orthopedic evaluation

A visual lameness evaluation (no lameness, mild lameness or minor gait abnormality, moderate lameness or obvious gait abnormality, severe weight-bearing lameness, non-weight-bearing lameness) and an evaluation of the dogs’ muscle condition (normal, atrophied) were carried out. The spine was palpated for a pain reaction (yes/no) in the neck, back, or LS area. The joints of the thoracic and pelvic limb were evaluated for pain, swelling, crepitation, instability, and decreased range of motion (yes/no). The stifles were additionally evaluated for possible patellar luxation (yes/no, and if yes, the grade on a scale from I–IV), as well as a positive tibial compression or cranial drawer tests (yes/no).

### Neurological examination

The neurological examination included evaluation of the dogs’ mental status, posture, gait, assessment of postural reactions, spinal reflexes including perineal reflex and bulbourethral reflex, and finally, evaluation of cranial nerves. A scale from 0 to 3 (absent, decreased, normal, increased) was used for evaluating postural reactions and spinal reflexes. The panniculus reflex (normal, absent, abnormal) was considered normal if absent in an otherwise neurologically healthy dog and abnormal if there was a cut-off point [21].

### Anesthesia

For anesthesia, the dogs received intramuscular premedication with 0.3 mg/kg methadone (Insistor vet® 10 mg/mL; Richter Pharma AG, Wels, Austria) and 0.02 mg/kg acepromazine (Plegicil® 10 mg/mL; Bela-Pharm GmbH & Co KG, Vechta, Germany). Propofol (Propovet Multidose 10 mg/mL; Fresenius Kabi AB, Uppsala, Sweden) was given 1–4 mg/kg to effect and anesthesia was maintained with sevoflurane (end-tidal concentration 2.3%) in oxygen.

### Electrodiagnostic studies

We performed all electrophysiological examinations with a Cadwell electrodiagnostic machine (Cadwell Industries, Inc., Kennewick, WA). EMG was performed bilaterally in the proximal and distal muscles of the pelvic and thoracic limbs, the paraspinal muscles and the perineal musculature. A disposable concentric needle electrode was used for EMG analysis and a subdermal needle electrode placed subcutaneously on the animal’s flank served as the ground. Abnormalities detected included abnormal insertional activity and any abnormal spontaneous activity such as fibrillation potentials and positive sharp waves.

MNCV of ulnar and peroneal nerves was measured unilaterally, performed with two stimulating monopolar needle electrodes. Subdermal needle electrodes served as a recording electrode, a reference electrode, and a ground electrode. Electrophysiological data were interpreted in comparison with published values [22–24]. The rectal temperature of the dog was measured during the electrodiagnostic testing.

### Computed tomography

Dogs underwent a CT scan using a helical 64-slice multidetector CT scanner (Lightspeed VCT, GE Healthcare, Madison, WI, USA) with a voltage of 120 kV, collimation pitch 0.516, speed 20.62 mm/rotation, rotation time 0.6 s, detector coverage 40 mm, matrix 512 × 512, and slice and interval thickness 0.625 mm. Using automodulation, the maximum current varied between 650 and 750 mAs. Dogs were in dorsal recumbency with hindlimbs in the “frog position” and scanned from the middle of the fourth lumbar vertebra to the most caudal aspect of the dog. Contrast agent 2 mL/kg (Omnipaque® 300 mg/mL) was injected into the *vena cephalica* using a power injector (Medrad® Stellant CT Injection System, Bayer AG, Leverkusen, Germany), and contrast scans were taken 1 min after the beginning of the injection.

CT images were assessed using a commercially available DICOM image processing workstation (Osirix®, v 11.0.4, Pixmeo, Switzerland) in MPR reconstruction. The lumbosacral spine was evaluated for intervertebral disc protrusion, as well as partial intervertebral foramen occlusion, using soft tissue windowing (window level 40, width 400). Any possible intervertebral disc protrusion was recorded and, if present, the disc space (L4-S1) and the percentage of protrusion into the spinal canal [none/minimal, slight (< 25%), moderate (25–50%), severe (> 50%)] were noted [25]. In the presence of protrusion, signs of compression such as any shift of the epidural fat or asymmetry in the shape of the spinal cord or cauda equina on transverse images were recorded. If these signs of compression were absent, the protrusion was always classified as slight and deemed clinically irrelevant [26]. In the case of partial occlusion of the intervertebral foramen, the severity (none, slight, moderate, and severe), type (soft tissue dense material, bone proliferation), disc space (L4-S1), and the location of the occlusion (left, right, or both) were assessed. The bony structures of the spine, pelvis, and hip joints were assessed using a bone window (window level 500, width 3500). The spine was assessed for vertebral anomalies and spondylosis, as well as signs of bone proliferation or irregularity of the sacroiliac joint (no/yes). The LS area was assessed for a visually obvious LS step between vertebral bodies (no/yes) and, if present, the step was measured (insignificant if < 2 mm, significant if > 2 mm) [25].

Hip joints were assessed for signs of degenerative joint disease by assessing signs of periarticular bone proliferation (osteophytes, enthesophytes), subchondral sclerosis, or incongruency. The findings (no lesions/femoral head and acetabulum congruent, developing/only presenting osteophytes at the dorsal acetabular rim or femoral head, established/presenting severe sclerosis and incongruency of the femoral head and acetabulum) were noted [27].

### Statistics

Statistical analysis was performed by a PhD researcher (T.Å.) using IBM® SPSS® Statistics, version 27.0.1 (IBM corp.©, Armonk, NY, USA). The findings from questionnaires and orthopedic or neurological evaluation were presented as a percentage (n/n), while continuous variables were presented as the mean (± standard deviation (SD); range) if normally distributed, otherwise as the median (interquartile range (IQR); range). The Pearson’s chi-squared test was used to explore possible etiological mechanisms. The following were tested: raw food diet and constipation, urinary incontinence and neurological or CT findings, CT findings and perineal- or bulbourethral reflexes. P-values less than 0.05 were considered statistically significant.

## Results

### Background information on the dogs and results from owner questionnaire

Altogether, 66 dogs met the inclusion criteria, representing 36 different breeds. The most common breeds were as follows: mixed breed (n = 9), Cotton de Tulear (n = 8), Dachshund (n = 5), Shetland Sheepdog (n = 3), Australian Kelpie (n = 2), Belgian Shepherd (n = 2), Bichon Frisé (n = 2), Border Collie (n = 2), Boxer (n = 2), Collie (n = 2), Finnish Lapphund (n = 2), and Norwegian Elkhound (n = 2).

The mean age (± SD; range) was 7.9 (± 1.8; range 4.6–11.6) years, and median weight 12.6 kg (IQR 15.1; range 4.2–38.0). Before being referred to the VTHUH, 10.6% (7/66) of the dogs were surgically castrated, with the mean age at castration being 6.5 (± 0.7; range 3.2–9.5) years. Although 89.4% (59/66) of dogs were still intact at the time of surgery, 19.7% (13/66) had received hormonal treatment with delmadinone acetate (10.6%, 7/66), osaterone acetate (7.6%, 5/66), or other (1.5%, 1/66), with the time of treatment ranging from 3 weeks to 12 months before surgery.

The most common type of diet, consumed by 53.0% (35/66) of dogs, was commercial, followed by a combination of commercial with raw (21.2%, 14/66), commercial with home-cooked (12.1%, 8/66), and a combination of the three (6.1%, 4/66). In 7.6% (5/66) of dogs, the diet was noted as “other”. Deworming medication was administered to 87.9% (58/66) of dogs, with a mean of 8.0 (± 13.1; range 0.6–97.0) months before the study.

**Fig. 1:**
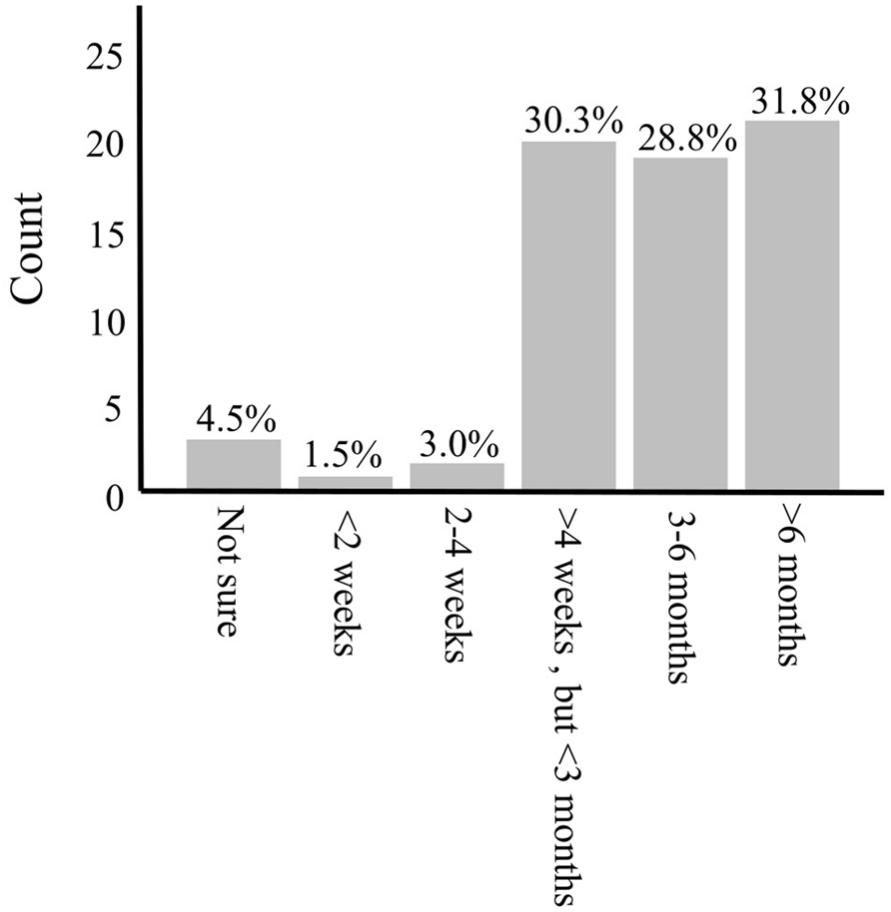
Time since presentation of the first signs associated with perineal hernia according to the owner questionnaire in 66 dogs with perineal hernia

The most common current GI sign in the owner questionnaire was “defecating small quantities at a time” reported by 87.9% (58/66) of owners, closely followed by bulging in the perineal area 86.4% (57/66) (Table 1). The duration of current GI signs varied, with 31.8% (21/66) being affected for over 6 months (Fig. 1). Urinary signs occurred less frequently, with urinary incontinence being the most common, affecting 12.1% (8/66) of dogs (Table 2).

**Table 1:** Current gastrointestinal signs according to the owner questionnaire in 66 dogs with perineal hernia

Sign	Present n (%)	Absent n (%)	Unsure n (%)
Inappetence	9 (13.6)	55 (83.3)	2 (3.0)
Vomiting	21 (31.8)	43 (65.2)	2 (3.0)
Diarrhea	20 (30.3)	45 (68.2)	1 (1.5)
Tenesmus	38 (57.6)	27 (40.9)	1 (1.5)
Defecation of small quantities	58 (87.9)	8 (12.1)	0 (0.0)
Problems evacuating the bowel	55 (83.3)	10 (15.2)	1 (1.5)
Staying in the defecation position for longer	56 (84.8)	10 (15.2)	0 (0.0)
Constipation	23 (34.8)	43 (65.2)	0 (0.0)
Thickened stool	24 (36.4)	40 (60.6)	2 (3.0)
Mucus in stool	11 (16.7)	49 (74.2)	6 (9.1)
Blood in stool	7 (10.6)	58 (87.9)	1 (1.5)
Fecal incontinence	6 (9.1)	60 (90.9)	0 (0.0)
Rectal prolapse	5 (7.6)	59 (89.4)	2 (3.0)
Bulging in perineal region	57 (86.4)	1 (1.5)	8 (12.1)

*n* number of dogs

**Table 2:** Current urinary signs according to the owner questionnaire in 66 dogs with perineal hernia

Signs	Present n (%)	Absent n (%)	Unsure n (%)
Pollakiuria	6 (9.1)	56 (84.8)	4 (6.1)
Stranguria	3 (4.5)	53 (80.3)	10 (15.2)
Dysuria	2 (3.0)	64 (97.0)	0 (0.0)
Urinary incontinence	8 (12.1)	57 (86.4)	1 (1.5)
Hematuria	1 (1.5)	64 (97.0)	1 (1.5)

*n* number of dogs

When evaluating chronic GI signs occurring before signs associated with PH, constipation was reported in 21.2% (14/66) (Table 3). There was no significant association between a raw food diet and constipation (P = 0.5). Chronic diarrhea was reported in 18.2% (12/66) and flatulence in 22.7% (15/66).

**Table 3:** Chronic gastrointestinal signs occurring before signs associated with perineal hernia according to the owner questionnaire in 66 dogs with perineal hernia

Signs	Present n (%)	Absent n (%)	Unsure n (%)
Vomiting	7 (10.6)	58 (87.9)	1 (1.5)
Diarrhea	12 (18.2)	54 (81.8)	0 (0.0)
Flatulence	15 (22.7)	45 (68.2)	6 (9.1)
Blood in feces	2 (3.0)	62 (93.9)	2 (3.0)
Mucus in feces	7 (10.6)	50 (75.8)	9 (13.6)
Constipation	14 (21.2)	44 (66.7)	6 (9.1)

*n* number of dogs

The most common previously diagnosed GI disease was food sensitivity (n = 7), followed by colitis (n = 1), proctitis (n = 1), IBD (n = 1) and tylosin-responsive diarrhea (n = 1). Other previously diagnosed diseases included prostatic hypertrophy or prostatitis (n = 14), anal gland impaction or inflammation (n = 9), hip joint laxity (n = 3), and benign perianal adenoma (n = 1). Neurological conditions had been diagnosed in four dogs, all with herniated intervertebral discs: one in the lumbar region, one in the cervical region, one with changes in both regions, and one not specified. No other degenerative lumbar conditions were reported. In addition, none of the dogs had been diagnosed with fractures.

### Orthopedic evaluation

In the orthopedic evaluation, a pain reaction was displayed by 13.6% (9/66) on palpation of the spine, with 6.1% (4/66) reacting to palpation of the LS area. Signs of generalized muscular atrophy were found in 7.6% (5/66). Mild lameness occurred in 9.2% (6/65) of dogs, with hind limb lameness in three.

In 31.8% (21/66) of dogs, palpatory findings occurred in one or more joint of the limbs. Crepitation and/or pain in one or more joints of the forelimbs was present in 16.7% (11/66). Of those dogs with hind limbs affected, 18.2% (12/66) had pain or a limited range of motion in one or both hips and 1.5% (1/66) had crepitation in the left hock. Bilateral patellar luxation was diagnosed in 12.1% (8/66) of dogs and unilateral in 6.1% (4/66), with a grade of I/IV in nine limbs, II/IV in ten limbs, and III/IV in one limb.

### Neurological examination

The most common neurological finding was an abnormal perineal reflex, missing in 7.7% (5/65) of dogs and decreased in 36.9% (24/65), as well as the bulbourethral reflex, missing in 3.1% (2/65) and decreased in 36.9% (24/66) of dogs. Both reflexes were normal in only 50.8% (33/65). The panniculus reflex had a cut-off point in 16.9% (11/65) of dogs (Table 4).

**Table 4:** Clinical findings in the neurological examination of 66 dogs with perineal hernia

Neurological signs	Abnormal n (%)	Normal n (%)
Mental status	0 (0.0)	66 (100.0)
Posture	1 (1.5)	65 (98.5)
Gait	2 (3.0)	64 (97.0)
Postural reactions	7 (10.6)	59 (89.4)
Spinal reflexes	1 (1.5)^a^	64 (98.5)^a^
Perineal reflex	29 (44.6)^a^	36 (55.4)^a^
Bulbourethral reflex	26 (40.0)^a^	39 (60.0)^a^
Cranial nerve examination	4 (6.2)^a^	61 (93.8)^a^
Panniculus reflex	11 (16.9)^a^	54 (83.1)^a^

*n* number of dogs

^a^Results missing in one dog

### Electrodiagnostic studies

Abnormal insertion potentials were present in 6.3% (4/64) of dogs, all within the perineal musculature. Three of these dogs also had abnormal spontaneous EMG activity in the perineal musculature. Only one dog had spontaneous EMG activity in another muscle group, namely the left front limb extensor carpi radialis. EMG was normal in 92.2% (59/64) of dogs. Ulnar MNCV was above reference values in all dogs, with a mean of 74.8 (± 15.6; range 45–158) m/s. Similarly, the peroneal MNCV was above reference values in all dogs, with a mean of 105.2 (± 21.8; range 65–155) m/s.

### Computed tomography

The most common CT finding was spondylosis deformans, observed in 24.6% (16/65) of dogs (Table 5). Partial occlusion of the intervertebral foramen at the LS junction was mild in 12.3% (8/65) and moderate in 6.2% (4/65) of dogs. In these dogs, partial occlusion was caused by spondylosis deformans in 83.3% (10/12) and soft tissue dense material in 16.7% (2/12). Partial occlusion was bilateral in 33.3% (4/12), left sided in 50.0% (6/12), and right sided in 16.7% (2/12). LS disc protrusion was present in 55.4% (36/65) of dogs, out of which 83.3% (30/36) of occurrences were slight and 16.7% (6/36) moderate. Disc protrusion in other than the LS disc space occurred in two dogs, one slight and one moderate. The types of vertebral malformation included incomplete fusion of the sacral crest (35.7%, 5/14), sacrocaudal fusion (42.9%, 6/14), or both (21.4%, 3/14). In 43.1% (28/65) of dogs, evaluated vertebral CT findings were absent. One dog had a visually obvious LS step which, when measured, was less than 2 mm and thus insignificant.

**Table 5:** Spinal column and hip joint findings in computed tomography images of 65 dogs with perineal hernia

Finding	Present % (n)	Absent % (n)
Moderate disc protrusion at the LS junction	9.2 (6)	90.8 (59)
Spondylosis deformans	24.6 (16)	75.4 (49)
Vertebral malformation	21.5 (14)	78.5 (51)
Partial occlusion of the intervertebral foramen	18.5 (12)	81.5 (53)
Visually obvious step at the LS junction	1.5 (1)	98.5 (64)
Sacroiliac joint degeneration	4.6 (3)	95.4 (62)
Hip-joint degeneration	21.5 (14)	78.5 (51)

*n* number of dogs, *LS *lumbosacral

No significant association was found between any of the assessed CT findings from the spine and abnormal perineal or bulbourethral reflexes (P > 0.1). However, a significant association was found between urinary incontinence and moderate disc protrusion at LS (P = 0.03).

Developing proliferative changes of the hip joint were found in 20.0% (13/65) and established in 1.5% (1/65) of dogs. Only two dogs with hip pain had proliferative changes of the hip joint. Out of the four dogs with pain on palpation of the LS area, one had osteophytes near the sacroiliac joint and three had spondylosis in the LS area, causing moderate occlusion of the intervertebral canal in one.

## Discussion

This is the most thorough study to date assessing preoperative data on dogs with PH. We applied methods not previously used to investigate PH etiology, such as orthopedic and neurological evaluations, as well as electrodiagnostic studies. In addition, we used CT imaging, a modality that, unlike radiography, allows for detailed evaluation of the pelvic area. Our findings suggest there is no evidence for association between PH and orthopedic conditions or a primary neuropathy. The neurological evaluation indicated nerve damage in the perineal area, but most likely secondary to PH rather than as a primary cause of the disease. In addition, based on the owner questionnaire, there was no evidence for association with GI diseases.

In accordance with previous studies, most dogs in our study were intact (89.4%), with castrated dogs having undergone the procedure at a mature age of four years or older. In addition, dogs that had received hormonal medication had been treated approximately 3–12 months before being referred, with prostatic disease cited as the most common reason for medication. The mean age and weight of the dogs were similar to findings in other studies [4, 8, 11]. Our study included 36 breeds, with mixed breed, Cotton de Tulear, and Dachshund being the most common. With such a broad spectrum of breeds and no control population available, evidence of breed predisposition remains undefined.

Previous studies have reported perineal bulging as well as straining to defecate as typical clinical signs of PH, which is in accordance with our findings [4, 14, 28]. Duration of clinical signs varies between studies, however, the only study that excluded dogs with previously operated PH had a mean duration of 2.5 months [28]. According to the owner questionnaire, dogs in our study had shown clinical signs related to PH for a significant period, exceeding 3 months in 60.6% of dogs. As PH related clinical signs often wax and wane, as well as slowly increase in severity, the exact time of PH formation and thus the distinction between signs related to the etiology of PH and those associated with PH is difficult.

Focal neurogenic atrophy as a causative factor for PH is one of the most popular theories in previous literature [2, 12, 29]. Similarly, in our study, by far the most common abnormality in the neurological examination of the dogs was a focal neurological deficit, namely, a decreased or absent perineal or bulbourethral reflex (49.2%). The perineal and bulbourethral reflexes test the pudendal nerve, as well as spinal cord segments S1 to Cd5 and their nerve roots. However, clinical signs that are often associated with lesions caudal to segment S1, including urinary and fecal incontinence, affected gait, and tail dysfunction, were uncommon in our study [21]. Furthermore, we found no evidence for an association between decreased perineal or bulbourethral reflexes and CT findings from the spine. A possible cause for abnormal perineal and bulbourethral reflexes could be local traction and/or stretching of the muscle and nerve structures in the perineal area, when no other causes for decreased reflexes were detected. We propose that this could be caused by pressure from the hernia content in PH dogs.

In a previous study using EMG on dogs with PH, an intraoperative assessment of separate perineal muscles was performed, revealing spontaneous activity in 35/40 dogs. These changes were indicative of denervation or primary muscle disease, but the authors simply focused on the perineal area [2]. In our study, MNCV measurement of the ulnar and peroneal nerves and EMG of the muscles of the limbs, paraspinal, and perineal musculature were conducted to assess possible generalized neuro- and myopathies [30]. In 92.2% of dogs, EMG findings were normal, and MNCV was within reference values in all dogs. Thus, no signs of generalized neuro- or myopathies were detected, and electrodiagnostic results also indicated that any denervation of the perineal muscles was caused by a local phenomenon, most probably being the effect of PH. In summary, neurological examination and electrodiagnostic findings in our study indicate possible nerve damage in the perineal area, probably occurring in the muscular branches of the pudendal nerve, which is in accordance with previous studies [2].

The focus of CT imaging was on the lumbosacral spine and hips. CT and magnetic resonance imaging have excellent agreement in detecting findings of LS stenosis [25], while radiography, used in previous PH studies, is insufficient for assessing spinal or intrapelvic changes [31]. Partial occlusion of the intervertebral foramen in 18.5% (12/65) of dogs was unlikely to cause nerve compression in the 66.7% (8/12) categorized as mild. The clinical relevance of moderate intervertebral foraminal occlusion and disc protrusion present in 6.2% (4/65) and 9.2% (6/65) of dogs, respectively, remains uncertain, but is unlikely to contribute to PH formation as these mild changes were not associated with neurological deficits. Mild hip joint degeneration and spondylosis deformans, conditions possibly causing pain while in the defecating position, were diagnosed in approximately 21.5% and 24.6% respectively. However, out of these only two with hip joint degeneration and three with spondylosis had pain on palpation of the affected area. In our population of mature dogs, this percentage of degenerative changes is to be expected and is probably unrelated to PH [26].

GI disease, such as colitis and proctitis, can cause straining, which is thought to predispose to PH [14]. Our questionnaire was designed to reveal possible chronic GI clinical signs that precede PH related clinical signs, being the first study to do so. In addition, it included questions on previously diagnosed GI diseases, which were noticeably few in our study group. Diarrhea, a common clinical sign associated with GI disease, was chronic in 18.2% of dogs. There are no previous studies evaluating GI signs occurring before signs associated with PH, however, concurrent diarrhea associated with PH has been reported in 14.7% (5/34) dogs to 32% (10/31) dogs [14, 28]. One study on 72 dogs with PH found diarrhea to present occasionally in 50% and frequently in 5%, and considered this an expected prevalence for hospitalized patients [4]. In our study, mucus and blood in the stool were reported as chronic signs in 10.6% and 3.0% of dogs, respectively. We suggest that these clinical signs were secondary to mucosal irritation in the colon and rectum caused by impacted feces, rather than GI diseases. Unfortunately, the exact time of PH formation remains undefinable, creating a challenge for questionnaire interpretation.

Chronic constipation is considered to increase intra-abdominal and perineal pressure and thus contribute to hernia formation. Constipation was reported as chronic in 21.2% of dogs and was thus more prevalent than diarrhea. A diet of raw food and/or bones can lead to constipation and straining, thus possibly contributing to PH [4]. However, in our study, no evidence for an association was found between a raw food diet and constipation. Whether chronic constipation was the cause of PH or a clinical sign of emerging PH is uncertain. In addition, considering that 66.7% of owners reported their dogs to be free of chronic constipation, it is unlikely to cause PH.

Urinary incontinence, the most common urinary sign in our study, occurred in 12.1% of dogs. Previous studies have reported urinary incontinence in 0–10% and urinary obstruction in 6–16.1% [6, 14, 28]. As urinary incontinence can be a clinical sign of LS stenosis [32], possible associations with neurological and CT findings were evaluated. An association was found between urinary incontinence and moderate disc protrusion, but due to the lack of other clinical signs, the significance of this finding is challenging to interpret.

The owner questionnaire, despite its careful construction, structure, and wording, was one of the major limitations in this study. The owners’ acuity in observing their dog and accuracy of their responses might have affected the collected data. The diagnosis of GI disease was based on the owner questionnaire, as more definitive results would have required biopsies, which was not within the scope of this study. As mentioned before, it is impossible to know the exact time of hernia formation and, as such, difficult to evaluate clinical signs that preceded it.

The lack of a control group of healthy intact male dogs limits our study to a descriptive one. In addition, sample size was relatively small, although our study being one of the largest prospective studies on PH. Dogs in our study group were often referred to the VTHUH from other clinics, which might have affected our selection, possibly excluding dogs with an urgent need for intervention, such as bladder retroflexion.

PH, as a disease of male dogs, is suspected to have a hormonal component, but no direct connection between sex hormones and PH has been detected [7]. The hormone relaxin, mainly produced in the prostate in male dogs, is thought to leak into surrounding tissue from a cystic prostate in PH dogs [9]. This study did not assess the prostate, as CT findings of the prostate were recently published in a separate study, which found PH dogs to have significantly larger prostates than age-matched males, often with cysts [20].

There are several differences between PH in humans and dogs. In dogs, PH is not uncommon and mainly affects intact male dogs, with no obvious etiological factors [4]. In human medicine, PH occurs most often secondary to urogenital or rectal surgical procedures, with primary PH being extremely rare. In primary PH, women are more often represented, with increased abdominal pressure from pregnancy, obesity, or ascites as a common risk factor [33]. Interestingly, there are some similarities between inguinal hernia in humans and PH in dogs, as both occur in the pelvic area, mainly affect older males, and have an obscure etiology. Due to the gender distribution of the inguinal hernia, a hormonal component is possible but has not yet been proven [34]. In dogs, the concurrent occurrence of inguinal hernia and PH has been reported [35, 36]. We found no evidence of inguinal hernia in the 66 dogs in our study, but a similar mechanism of disease is possible.

## Conclusion

Our study did not provide evidence that orthopedic, neurological, or GI diseases contribute to PH formation, confirming our hypothesis. Future studies should focus on the role of the prostate and its possible contribution to PH through the hormone relaxin or other possible paths.

## Data Availability

The datasets used and/or analyzed during the current study are available from the corresponding author on reasonable request.

## Abbreviations

CT: Computed tomography
EMG: Electromyography
GI: Gastrointestinal
LS: Lumbosacral
MNCV: Motor nerve conduction velocity
PH: Perineal hernia
VTHUH: Veterinary Teaching Hospital of the University of Helsinki
